# Using interpersonal affect regulation in simulated healthcare consultations: an experimental investigation of self-control resource depletion

**DOI:** 10.3389/fpsyg.2015.01485

**Published:** 2015-09-29

**Authors:** David Martínez-Íñigo, Francisco Mercado, Peter Totterdell

**Affiliations:** ^1^Department of Psychology, Universidad Rey Juan Carlos Madrid, Spain; ^2^Department of Psychology, Sheffield University Sheffield, UK

**Keywords:** affect regulation, self-regulation, conservation of resources, ego depletion, performance, emotion, healthcare

## Abstract

Controlled Interpersonal Affect Regulation –the process of deliberately influencing the internal feeling states of others– occurs in a variety of interpersonal relationships and contexts. An incipient corpus of research shows that interpersonal affect regulation can be characterized as a goal-directed behavior that uses self-control processes which, according to the strength model of self-regulation, consumes a limited resource that is also used by other self-control processes. Using interpersonal affect-improving and affect-worsening regulation strategies can increase agent’s resource depletion but there is reason to think that effects will partially rely on target’s feedback in response to the regulation. Using a healthcare paradigm, an experiment was conducted to test the combined effects of interpersonal affect regulation use and patient feedback on healthcare workers’ resource depletion, measured as self-reported experienced and expected emotional exhaustion, and persistence on a self-regulation task. Medical students (*N* = 78) were randomly assigned to a 2(interpersonal affect regulation: affect-worsening vs. affect-improving) × 2(patients’ feedback: positive vs. negative) factorial between-subjects design and given instructions to play the role of doctors in interactions with two professional actors trained to act as patients. Analysis of covariance showed that affect-worsening was more depleting than affect-improving for all measures, whereas the recovery effects of positive feedback varied depending on strategy type and measure. The findings confirm the characterization of interpersonal affect regulation as potentially depleting, but suggest that the correspondence between the agent’s strategy and the target’s response needs to be taken into consideration. Use of affect-improving and positive feedback showed positive effects on self-rated performance, indicating that interpersonal affect regulation is relevant for organizational as well as personal outcomes.

## Introduction

When doctors try to make patients feel less distressed about their health diagnosis, or call center operators attempt to make customers feel regret for not accepting an “incredible” offer, or flight attendants act to calm down anxious passengers, they are engaging in a process of controlled *interpersonal affect regulation* because they are deliberately trying to influence the internal feeling states of another person, including their moods and emotions. Controlled interpersonal affect regulation is a common phenomenon in interpersonal interactions (Parkinson et al., 2005) that contributes to social coordination and communication and motivates behaviors, including those found in the workplace (Niven et al., 2009).

Niven et al. (2009) have empirically established two major categories of interpersonal affect regulation. First, they define interpersonal affect regulation that is aimed at improving how others feel *(affect-improving)*. This can involve someone (the agent) trying to induce a positive feeling in a target person but it can also involve reducing a negative feeling. Examples of affect-improving strategies are paying clients compliments and listening to their problems. A second category describes interpersonal affect regulation that is aimed at worsening how others feel *(affect-worsening)*. This can involve trying to induce a negative feeling in a target person but it can also involve dampening a positive feeling. Examples of affect-worsening strategies are making defaulting clients aware of the negative consequences that their behavior may have upon the financial situation of their family or withdrawing attention from clients to extinguish their unwarranted demands.

In the context of work, employee’s regulation of other’s emotions has been shown to be relevant for their own well-being and for the achievement of organizational goals. Attempts to change client’s affect can have an impact on employees’ own well-being (Niven et al., 2012b; Martínez-Iñigo et al., 2013). Use of interpersonal affect regulation strategies has also been related to interpersonal processes that are highly relevant for organizational outcomes. Niven et al. (2012a), for example, found that worker’s interpersonal affect regulation influenced the quality of their relationships at work. This association is relevant for organizations because employees’ competency in developing positive relationships at work has strategic value for many organizational activities, especially for organizations in the service sector where the primary business activity concerns social interactions with clients (Adler and Kwon, 2002; Chou et al., 2008). Despite empirical evidence demonstrating the relevance of interpersonal affect regulation for employee’s well-being and organizational outcomes, research on interpersonal affect regulation in the workplace is scant especially when compared with the amount of evidence that has been compiled concerning the consequences of employee’s regulation of their own emotions (for a review, see Hülsheger and Schewe, 2011).

Existing evidence concerning the effects of interpersonal affect regulation relies predominantly on survey study designs which threatens its validity because such designs are unable to establish causal relations between variables with any certainty. Two studies have experimentally tested the consequences of interpersonal affect regulation for well-being but both only focused on affect-improving strategies (Study 1 in Martínez-Iñigo et al., 2013, Study 2 in Niven et al., 2012b). One of these studies was followed by a longitudinal survey study of healthcare workers (Study 2 in Martínez-Iñigo et al., 2013) which found that the effect of interpersonal affect strategies on emotional exhaustion (an aspect of well-being concerning resource depletion) depended on the nature of feedback that healthcare workers received from clients, but this effect has yet to be demonstrated experimentally. Feedback from clients may vary because interpersonal affect regulation strategies are not equally effective in meeting their demands or for other reasons, either of which could influence workers’ perceived performance but again this has not been tested. Relevant research on worker’s regulation of their own emotions (rather than other peoples’ emotions) during client interactions has shown that the type of strategy employees use contributes to how well they perceive they have performed (Brotheridge and Grandey, 2002; Humphrey et al., 2015).

Consequently, the aims of the current study were twofold: (1) to strengthen the empirical evidence linking interpersonal affect regulation to resource depletion, by experimentally testing the joint effects of agents’ use of interpersonal affect regulation strategies and targets’ feedback on two self-reported measures (experienced emotional exhaustion and expected emotional exhaustion) and one behavioral indicator (persistence on a self-control task) of resource depletion, and (2) to provide evidence on the connections between the use of interpersonal affect regulation strategies and perceived performance in a service encounter. These aims were examined in the context of a healthcare simulation in which medical doctors were consulted by patients about a health problem. Although regulation of other’s affect is a pervasive characteristic in service delivery interactions, its study in the context of physician-patient encounters has especial implications due to its relevance to obtain positive outcomes in healthcare provision (Finset and Mjaaland, 2009). Research has consistently shown that physician’s skills to deal with the patient’s positive and negative emotions (e.g., being warm and friendly or firm) are related to better health outcomes measured physiologically (blood pressure), behaviorally (functional status), or self-reportedly (patient overall perception of their health; Kaplan et al., 1989). Physician-patients encounters where the physician takes care of the patient’s emotions are significantly more effective than neutral interactions in reducing the patient’s suffering (Di Blasi et al., 2001; Larson and Yao, 2005). There is also some evidence on the relevance of emotional care for organizational outcomes. The frequency of patient’s attendance to health services decreases and the speed of recovery increases when health professional care about the patient’s emotions (Redelmeier et al., 1995; Di Blasi et al., 2001). All these evidence support the assumption that physician’s skills to regulate patient’s affect contributes to the effective delivery of health care. The experimental study of interpersonal regulation of affect in the context of simulated physicians-patient consultations complements previous research on physician-patient interaction more focused on the health outcomes for the patients and emphasizes the practical implications of our study.

### Interpersonal Affect Regulation and Emotional Exhaustion

#### Experienced Emotional Exhaustion

Emotional exhaustion is an important aspect of employee’s affective well-being that refers to feelings of being overextended and depleted of one’s emotional resource (Maslach, 1993; Maslach et al., 1996; Van Horn et al., 2004). Employees who are continuously exposed to service encounters that demand the regulation of their own and other’s emotions may end up in a state of ego-depletion (Muraven and Baumeister, 2000). Ego-depletion is characterized as a state in which employee’s self-control resources have been drastically reduced by the sustained exertion of self-control, and their capacities to perform subsequent self-regulation tasks are seriously compromised (Muraven et al., 1998). Previous research has measured emotional exhaustion as an indicator of employee’s level of ego-depletion (Goldberg and Grandey, 2007). Feelings of being emotionally exhausted motivate individuals to protect or replenish their personal resources to avoid becoming totally depleted (Cropanzano et al., 2003). Empirical findings support the view that people’s regulation of their own emotions and other people’s emotions are significantly related to their level of emotional exhaustion (Niven et al., 2012b; Martínez-Iñigo et al., 2013).

The impact of employees’ affect regulation on their emotional exhaustion is thought to depend on the balance between two opposing processes (Côté, 2005; Lilius, 2012). The first process is the consumption of self-regulation resource through changing affect. Drawing on the strength model of self-regulation (Baumeister et al., 1998), affect regulation can be categorized as a deliberate self-regulation process that consumes self-regulation resource. In the case of interpersonal affect regulation, self-regulation is also required because it is a goal-directed activity that is directed at changing another person’s affect and demands self-control resource to do so. Employees are required to put some self-regulatory effort into the regulation of client’s affect during service encounters (see Webb et al., 2012). For instance, employee’s might need to: select which regulation strategy is most appropriate to the characteristics of the client; or monitor the deployment of the selected strategy; or detect when a change in the strategy is necessary to reach the goal. So interpersonal affect regulation can drain the individual’s limited self-regulation resource which may result in a state of *ego-depletion*. Affect-improving and affect-worsening are both conscious strategies that require agent’s self-regulation effort, but empirical evidence shows that they have different consequences for employee’s affective well-being. Niven et al. (2012b) found that trying to worsen other’s affect had a negative impact on the agent’s affective well-being, whereas the use of affect-improving strategies was positively associated with affective well-being.

The second process involved in the explanation of the effects of interpersonal affect regulation on emotional exhaustion is related to the use of opportunities to recover the self-regulation resource consumed in regulating the target’s affect. According to the Conservation of Resource Model (Hobfoll, 1989), client’s feedback during service encounters will be one of the main mechanisms that enables employees to recover this resource. Therefore, the target’s feedback in response to different interpersonal affect regulation strategies will contribute to explaining their consequences for employee’s emotional exhaustion. Previous research has shown that, in a natural context, agent’s interpersonal affect regulation strategies and target’s feedback are not independent (Martínez-Iñigo et al., 2013). The likelihood of receiving positive feedback from targets decreases when agents intentionally try to worsen the target’s affect, which increases the risk of agents becoming emotionally exhausted. In contrast, positive responses from targets are more likely when agents intentionally try to improve target’s affect, which counteracts the depleting effects of regulating the target’s affect.

Research on emotional labor has indirectly shown that the balance between the amount of self-regulation resources a specific strategy depletes and the quantity of resources it recovers through the interaction partner’s feedback contributes to explaining the effects of affect regulation on ego depletion (Brotheridge and Lee, 2002; Goldberg and Grandey, 2007). For interpersonal affect regulation, results show that healthcare worker’s (e.g., general practitioners, mental health workers) emotional exhaustion was greater after trying to worsen patient’s affect (Martínez-Iñigo et al., 2013). No relationship was found between using affect-improving strategies and emotional exhaustion, until feedback from the patient was controlled which produced a positive relationship. These results have been interpreted as indirect evidence that using affect-improving strategies produces positive feedback that buffers the draining effect. According with this rationale it is expected that the use of affect-improving strategies will not produce emotional exhaustion when it is followed by positive feedback, which contributes to replenishing the depleted self-regulation resource.

Drawing on these results we hypothesize that participants who use affect-improving strategies will report significantly less emotional exhaustion than participants who use affect-worsening strategies if they receive positive feedback, but will not differ if they receive negative feedback (H1a).

#### Expected Emotional Exhaustion

Research demonstrates that people’s expectancies about the energy that they will have available for future acts of self-control have an impact on their actual level of ego-depletion in a subsequent self-control task. Individuals’ expectancies are based on their beliefs about the limits of their self-control resource and their estimate of the amount of self-control resource that is demanded by current tasks (Martijn et al., 2002; Boucher and Kofos, 2012). Job et al. (2010) found that when participants believed that self-control resource was unlimited, there was no difference in their level of ego-depletion regardless of whether or not the previous task demanded self-control. Likewise, Martijn et al. (2002) showed that challenging people’s beliefs that previous acts of self-control drained their resources reduced their level of ego-depletion. In a similar vein, it has been demonstrated that having an expectation that self-control efforts will be accompanied by an alternative resource gain can ameliorate the effect of that effort on ego-depletion (Boucher and Kofos, 2012).

In line with these results, we expect that participant’s experience of interpersonal affect regulation use during interaction with the target will inform their expectations of how exhausting a series of future interactions with other targets are likely to be. The experience of using affect-improving strategies will heighten participants’ expectation that they will have sufficient resources available after future interactions, when compared to those who use affect-worsening, but only if they also receive positive feedback from the target. In natural contexts, the implementation of affect-improving strategies is positively related to target’s positive feedback (Martínez-Iñigo et al., 2013) and to the agent’s affective well-being (Niven et al., 2012b). In the absence of a source of resource recovery, such as positive feedback, individuals trying to improve another person’s affect may believe that the amount of available self-regulation resource has been decreased without being replenished through the target’s positive feedback and thus may expect that repeated interaction under this condition will be emotionally exhausting. We hypothesize that participants who use affect-improving strategies will expect to experience less emotional exhaustion than participants who use affect-worsening strategies if they receive positive feedback from targets, but will not differ if they receive negative feedback (H1b).

#### Interpersonal Affect Regulation and Persistence on a Self-Control Task

Numerous studies of self-control and ego-depletion have suggested that performance on a task demanding self-control can be impaired when it is attempted shortly after the execution of a previous self-control task (Hagger et al., 2010). The depletion of self-regulation resource on the execution of the first task reduces the amount of self-regulation resource available to perform the second and explains its impaired performance (Muraven and Baumeister, 2000). These studies are based on the strength model of self-regulation and typically employ a dual-task paradigm to test the self-regulation nature of a task (Baumeister et al., 1998). Under this paradigm participants assigned to the experimental condition are required to engage in two consecutive tasks demanding self-regulation. For the control group, only the second task requires self-regulation effort. According with the strength model of self-regulation, performance on the second task will be worse for the participants in the experimental group because of the resource they depleted performing the first self-regulation task (Baumeister et al., 2007; Tyler and Burns, 2008). Execution of a self-regulation task within one area (e.g., suppression of emotional expression) impairs performance in a different area (e.g., controlling attention or impulse control) because all types self-regulation rely on the same limited self-regulation resource (Muraven and Baumeister, 2000; Gailliot et al., 2007). Based on this research, we expected that interpersonal affect regulation, which is a task that demands self-regulation, would reduce performance on a subsequent self-control task. Under the dual-task paradigm, reduced persistence on the second task is considered a measure of performance impairment (Hagger et al., 2010).

As described for the effects on emotional exhaustion, the amount of resource left following interpersonal affect regulation is related to the amount of self-regulation resource consumed by the use of the interpersonal regulation strategy and the amount recovered through target’s feedback. Martínez-Iñigo et al. (2013) found that individual’s ego-depletion –measured by persistence on an unsolvable anagram– was greater when participants were required to improve the other person’s affect compared with encounters where interpersonal affect regulation was not demanded (Martínez-Iñigo et al., 2013, Study 1). In accordance with a conservation of resource model, the negative effects of previous self-regulation efforts on subsequent self-control can be ameliorated when individuals recover the depleted self-regulation resource thought positive feedback. Therefore we hypothesize that participants who use affect-improving strategies will persist significantly longer on a subsequent self-control task than participants who use affect-worsening strategies if they have received positive feedback from targets, but will not differ if they have received negative feedback (H2).

#### Interpersonal Affect Regulation and Performance

Research on employee’s self-regulation of their own emotions has shown that use of affect regulation is related to their perceptions of personal accomplishment and self-efficacy (Brotheridge and Grandey, 2002; Humphrey et al., 2015), both of which are directly related to performance (Van Eck Peluchette, 1993) and indirectly to the achievement of organizational results (Stajkovic and Luthans, 1998; Judge and Bono, 2001). Brotheridge and Grandey (2002) found that employees’ regulation of their own emotions significantly predicted their perceptions of how effectively they treated customers. However, evidence on relations between the use of different interpersonal affect regulation strategies and performance is absent.

Research on self-regulation suggests that relationships between ego depletion and perceptions of concurrent performance and future performance may have different explanations. It has been proposed that subjective experiences during a self-regulation task (e.g., a strong urge to eat a cookie) can influence assessments of performance, even when individuals succeed in a purely behavioral sense (Wallace and Baumeister, 2002). In assessing future performance, a reduction in self-regulation resource following the execution of a self-control task has been proposed to result in a perception of having diminished capacity to perform a subsequent self-regulation task (Hagger et al., 2010). However, empirical studies have found no evidence of ego-depletion effects on self-efficacy, or for meditational role of self-efficacy in the impairment of individual’s subsequent performance in the dual-task paradigm (Wallace and Baumeister, 2002; Baumeister et al., 2006; Gailliot and Baumeister, 2007). A possible explanation for this null finding is that reduced self-efficacy in one sphere may not necessarily spill over to perceptions of self-efficacy in another sphere, as in the dual task paradigm (Hagger et al., 2010).

From an interpersonal perspective, Finkel et al. (2006) found that social coordination may require different levels of self-regulation effort during interpersonal interaction. When participants are able to smoothly align their behaviors with one another, social coordination takes places in an effortless manner and thus interaction is defined as low maintenance. In contrast, when social coordination demands a lot of effort –thus draining a considerable amount of self-regulation resource– interaction is defined as high-maintenance. Coordinated social interaction typically takes place in an effortless manner (i.e., low maintenance) without interfering in the performance of other tasks (Hatfield et al., 1994). Nevertheless, despite being relatively uncommon, high-maintenance interactions can have an identifiable negative effect on individuals’ performance on other tasks. Finkel et al. (2006) found that high-maintenance interactions had a significant negative effect on participant’s performance on a second self-regulation task, measured with a variety of behavioral indicators (including performance difficulty of the task selected, number of problems correctly solved, and impaired task performance). When studying interpersonal problem-solving situations, Finkel et al. (2006, Study 4) found that an interaction partner’s failure to positively reciprocate the suggestions and advice offered by the agent resulted in impairments to the agent’s performance on a handgrip persistence task.

Social coordination is a core instrument for the achievement of goals in service delivery interactions so we expect that the observed effects of social coordination on ego-depletion will also influence individuals’ assessment of their performance. Specifically we expect that participants’ self-reported performance will be related to coordination between their interpersonal affect regulation strategy use and the feedback they receive from targets. Agents attempts to worsen target’s affect or targets’ negative responses to attempts to improve their affect will demand higher maintenance than interactions in which agent’s attempts to improve target’s affect are positively reciprocated. We propose that higher maintenance will be processed by agents as signals of poor performance because service encounters demand effective social coordination.

Projecting this rationale onto the current experiment, we hypothesize that participants in the experimental condition that requires low maintenance interaction (i.e., affect-improving with positive feedback) will rate their performance higher than participants in the conditions that require high maintenance interactions (i.e., affect-improving with negative feedback, affect-worsening with positive feedback, and affect-worsening with negative feedback) (H3).

## Materials and Methods

### Experimental Design

Participants were randomly assigned to a 2 (interpersonal affect regulation: affect-worsening vs. affect-improving) × 2 (patients feedback: positive vs. negative) factorial between-subjects design. Cell frequencies varied between 18 and 21. The order of presentation of the four conditions depended on the participants’ availability to attend the programmed experimental session.

### Participants

Participants were recruited through adverts posted on classroom noticeboards in a large University in the central region of Spain asking for medical students to participate in a study of the skills required for effective interaction with patients. Financial compensation (20€) was offered in exchange for participation in a session lasting 45–60 min. A total of 83 undergraduate medical students (58 women and 25 men) were recruited to the study. Participants had a mean age of 20.3 years (*SD* = 3.4) and were in different years of their medical studies. Data from two participants were lost because one of the confederates performed the wrong instructions for the condition and data from three participants were excluded because they did not complete the ego-depletion measures. The study was approved by the hospital’s research ethics committee.

### Measures

#### Experienced Emotional Exhaustion

Emotional exhaustion was measured using the Spanish version (Seisdedos, 1997) of the Maslach Burnout Inventory (MBI; Maslach et al., 1996) and was used as a self-reported indicator of resource depletion (Goldberg and Grandey, 2007). Participants were asked to report how emotionally exhausted they felt after the interaction with the simulated patients. The scale included five items (e.g., “After the consultation with the patients I feel emotionally drained”) measured on a 7-point response scale ranging from 0 (*never*) to 6 (*every day*). The internal consistency of the scale was α = 0.93.

#### Expected Emotional Exhaustion

Using the same instrument used to measure experienced emotional exhaustion, participants were asked to estimate how emotionally exhausted they would feel if they had to perform the same kind of interaction that they experienced during the experimental session for the whole working-day. The internal consistency of the scale was α = 0.93.

#### Anagram Persistence

The amount of time a participant spent trying to solve an unsolvable anagram was used as a behavioral indicator of ego-depletion (Segerstrom and Nes, 2007). The task was described to participants as a measure of individual differences that would help the researchers understand how individuals differed on the previous task. A solvable anagram (in Spanish) was presented first (*bedtarli*), followed by the unsolvable anagram (*rielojqu*) and then a third, solvable one (*aluja*). For ethical reasons, 15 min was set as the maximum time for persistence on the unsolvable anagram. The assistant required the six participant still working at this point to proceed on the next anagram and recorded their time as 15 min.

#### Self-rated Performance

This was measured with five items developed for the present study concerning how well participants considered they had performed in treating the patient (“I was able to easily handle face to face patient interactions”; “The way I have treated the patient has been efficient”; “I have met the patient’s demands”; “My performance has been professional”; “The patient felt understood”). Participants were asked to rate the items on a 7-point response scale from 0 (*Not at all*) to 6 (*Totally*). The internal consistency of this scale was α = 0.88.

#### Patient’s Feedback

To check the experimental manipulation of confederate’s feedback, participants were asked to rate the patient’s behavior toward them using three 7-point bipolar scales (*unpleasant-pleasant*, *frustrating-motivational* and *satisfactory-unsatisfactory*). The internal consistency for the scale was α = 0.81.

#### Control Variables

Individuals high in public self-consciousness have been found to be more persistent on anagram tasks (e.g., Nes et al., 2005). To ensure that anagram persistence measured self-regulation resource depletion (rather than simply the tendency to perform well in front of other people), the effect of public self-consciousness was controlled when estimating the effects of interpersonal regulation strategies and feedback on anagram persistence. This variable was measured with the seven item public self-consciousness subscale of the Self-Consciousness Scale (Fenigstein et al., 1975). Participants were required to rate how characteristic each of the seven statements were of them (e.g., “I’m concerned about my style of doing things” and “I usually worry about making a good impression”) on a 5-point scale from 0 (*extremely uncharacteristic*) to 4 (*extremely characteristic*). The internal consistency of the scale was α = 0.78. Additionally, we also controlled for the effect of gender.

### Procedure

A research assistant informed the participants that the study was concerned with the professional skills that are used to deal with patients and their effects on patient’s satisfaction and assessment of medical effectiveness. The research assistant was trained to conduct the study but was unaware of the hypotheses and specific goals of the study. Participants were informed that they would be role-playing a couple of medical consultations with two simulated patients who had been trained to assess the professionalism with which particular skills to manage patients are enacted. The assistant also informed participants that they would be asked to complete a questionnaire to gather information on their attitudes, aptitudes, and behaviors.

Once participants had agreed that they understood the process and had given informed consent, they filled out a questionnaire that contained measures of the control variables. To make sure that participants kept to the script for their assigned condition, they were given the following instruction: “We are interested in your ability to enact specific skills when interacting with patients. There are other effective skills that you are probably able to perform, but we are not interested in those. Please, do keep to the script we will describe to you.”

Half of the participants were instructed to perform skills reflecting the six affect-worsening strategies identified by Niven et al. (2011) and received the following instructions: “In order to be an effective professional and deliver a good quality service, a doctor needs to be able to make patients notice some negative aspects of his/her behavior and make them feel worse. This can be done through different skills. Below you will find a list of them. During the consultation we expect you to perform these skills.”

The other half of the participants were instructed to perform skills reflecting the six affect-improving strategies identified by Niven et al. (2011) as follows: “In order to be an effective professional and deliver a good quality service, a doctor needs to be able to psyche patients up and make them feel better. This can be done through different skills. Below you will find a list of them. During the consultation we expect you to perform some of these skills.”

All participants read through the list of skills corresponding to their condition. To check participants understood the instructions, the assistant asked a couple of simulated patient demands (e.g., the assistant showed doubts on the effectiveness of the treatment) the participant had to respond using some of the skills contained in the list (e.g., participant tried to make assistant laugh to improve the assistant’s affect or participant complains to the assistant about their behavior to try make the assistant feel worse) and gave the participants the opportunity to resolve any doubts they had about the instructions. If participants failed to perform one of the strategies on the assigned condition, the assistant pointed one of the correct responses, asked the participant to perform it and asked a new simulated patient demand. Once the participants solved correctly two demands they proceed with the session.

To increase the realism of the situation, participants were asked to wear signs of their job status and role (a laboratory coat and a phonendoscope) and were located in a position that differentiated them from the patients. The laboratory room used for the study was one of the surgeries in the university clinic where real patients usually attended. It was equipped with a two-way mirror and intercom for research and teaching purposes. The room was arranged as a standard primary care surgery (an interview area and an exploration area) and common elements of a surgery were incorporated (e.g., a prescription pad) including room decorations (e.g., health promotion posters).

The assistant asked the participant to read the simulated medical records of each patient before receiving them and then left. Simulated patients were enacted by a female and a male professional actor, who were unaware of the interpersonal affect regulation condition assigned to participants. The confederates were trained to follow an interactive script during the surgery which revealed their reason for seeking medical help and requested a medical prognosis and recommendations for treating their health problem. Additionally they were instructed to introduce conversation topics (e.g., the consequences of their condition for other people, difficulties in adhering to the treatment, and low expectations for their recovery) that gave the participants the opportunity to perform interpersonal affect regulation strategies corresponding to the experimental condition.

The confederates were instructed on how to give structured feedback for both positive and negative feedback conditions. Each confederate memorized a different set of three positive feedback and three negative feedback sentences to insert into the consultation according to the feedback condition (positive or negative) in response to the participant’s attempts to change his or her affect. After the first actor left the room, the second actor entered. The order of presentation of the two simulated patients within each experimental session was counter-balanced for each condition. Both confederates were instructed to close the interaction after 5 min of interaction.

After the role-play, participants were required to complete a series of three anagrams presented one at a time, the second of which was unsolvable. Participants were told that once they had solved an anagram or wanted to move on to the next (i.e., if they could not find the solution) they should inform the assistant observing from the control room by raising their hand and then proceed to solve the following anagram. Upon presentation of the unsolvable anagram, the assistant began timing how long participants persisted before giving up and requesting the next anagram. After the third anagram, participants were asked to fill out a questionnaire containing the self-report measures. After all data collection had finished, participants attended an appointment to receive financial compensation and were debriefed about the study.

### Analysis

To test the interaction effects contained in the hypotheses, we conducted a series of 2 (interpersonal affect regulation: affect-worsening vs. affect-improving) × 2 (feedback: negative vs. positive) between-subject analyses of covariance (ancova) with gender as a covariate for the self-reported measures, and gender and public self-consciousness as covariates for the behavioral measure of ego-depletion.

## Results

Means and standard deviation for each cell of the 2 × 2 design for the four dependent variables are shown in **Table 1**. Anagram persistence scores were transformed using a logarithm function to meet normality assumptions.

**Table 1:** Means and standard deviations for the affect regulation × feedback model.

		Affect-worsening		Affect-improving	
		Negative feedback	Positive feedback	Negative feedback	Positive feedback
Experienced E. exhaustion	*M*	1.54	1.59	1.66	0.50
	*SD*	0.32	0.27	0.22	0.17
Expected E. exhaustion	*M*	3.60	3.20	2.93	1.90
	*SD*	0.34	0.33	0.21	0.29
Log anagram persistence	*M*	2.41	2.42	2.62	2.45
	*SD*	0.06	0.05	0.07	0.08
Self-rated performance	*M*	2.09	2.59	3.23	4.46
	*SD*	0.24	0.28	0.21	0.17

### Experimental Checks

An independent samples *t*-test was conducted to compare perceptions of patients’ feedback between participants who received positive feedback and those who received negative feedback. As expected, participants in the positive feedback conditions, *M* = 4.79 (*SD* = 0.18), perceived the patients’ responses as being more positive, *t*(76) = -9.73, *p* = 0.001, than participants in the negative feedback condition, *M* = 2.75 (*SD* = 0.11).

### Experienced Emotional Exhaustion

**FIGURE 1:**
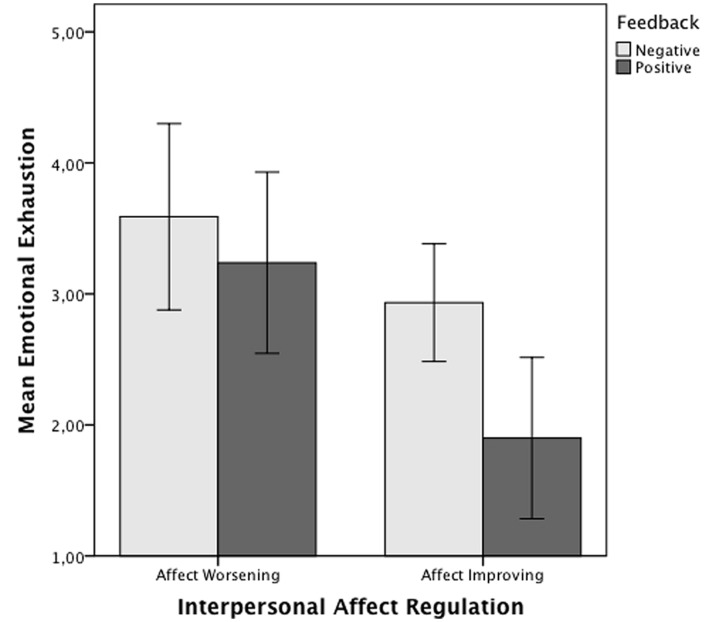
**Mean ratings of experienced emotional exhaustion for interpersonal affect regulation strategy and feedback conditions**. Error bars represent 95% confidence interval.

As predicted (H1a), a two-way ancova for emotional exhaustion indicated a significant interaction between agent’s interpersonal affect regulation strategy and target’s feedback, *F*(1,73) = 6.61, *p* = 0.01, $\eta_{p}^{2}$ = 0.08 (see **Figure 1**). Two Fisher’s LSD *post hoc* tests were conducted to examine the difference in the effect of positive and negative feedback, first, in the affect-improving condition and, second, in the affect-worsening condition. *Post hoc* multiple comparisons showed that affect-improving participants who received positive feedback reported significantly, *F*(1,73) = 12.36, *p* = 0.001, $\eta_{p}^{2}$ = 0.14, Cohen’s *d* = -1.31, lower emotional exhaustion, *M* = 0.50 (*SD* = 0.76), than affect-improving participants who received negative feedback, *M* = 1.66 (*SD* = 1.01), whereas for affect-worsening the difference between positive feedback, *M* = 1.59 (*SD* = 1.27) and negative feedback, *M* = 1.54 (*SD* = 1.35) was not significant, *F*(1,73) = 0.01, *p* = 0.91, $\eta_{p}^{2}$ = 0.00, Cohen’s *d* = 0.04. Hypothesis 1A was therefore supported.

### Expected Emotional Exhaustion

As might be expected, participant’s estimation of their emotional exhaustion after performing the same kind of interaction that they experienced during the experimental session for the whole working-day, *M* = 2.93 (*SD* = 1.42) was significantly higher, *t*(77) = -13.56, *p* = 0.001, than the emotional exhaustion they reported after the interaction with the two actors, *M* = 1.35 (*SD* = 1.20). Contrary to expectation (H1b), the interaction between the agent’s interpersonal affect regulation strategy and target’s feedback was not significant for expected emotional exhaustion, *F*(1,73) = 1.73, *p* = 0.19, $\eta_{p}^{2}$ = 0.02, so Hypothesis 1 b was not supported. However, the main effect for affect regulation strategy was significant, *F*(1,73) = 4.13, *p* = 0.001, $\eta_{p}^{2}$ = 0.02, with affect-improving participants reporting lower levels of expected emotional exhaustion, *M* = 2.46 (*SD* = 1.21) than affect-worsening participants, *M* = 3.40 (*SD* = 1.47). The main effect of target’s feedback was also significant, *F*(1,73) = 6.41, *p* = 0.02, $\eta_{p}^{2}$ = 0.08. Participants receiving target’s positive feedback, *M* = 2.62 (*SD* = 1.53) reported lower levels of expected emotional exhaustion than participants receiving negative feedback, *M* = 3.24 (*SD* = 1.24). As for experienced emotional exhaustion, *post hoc* analysis showed that the difference for expected emotional exhaustion between positive feedback, *M* = 3.24 (*SD* = 1.52) and negative feedback, *M* = 3.60 (*SD* = 1.42), in the affect-worsening condition was not significant, *F*(1,72) = 0.64, *p* = 0.42, $\eta_{p}^{2}$ = 0.01, Cohen’s *d* = 0.2, whereas in the affect-improving condition participants receiving targets’ positive feedback, *M* = 1.90 (*SD* = 1.24), reported significantly, *F*(1,72) = 6.89, *p* = 0.01, $\eta_{p}^{2}$ = 0.09, Cohen’s *d* = 0.2, lower expectations on future emotional exhaustion than participants in the negative feedback condition, *M* = 2.93 (*SD* = 1.00).

### Anagram Persistence

The interaction between interpersonal affect regulation strategy and feedback was not significant for anagram persistence, *F*(1,73) = 1.97, *p* = 0.16, $\eta_{p}^{2}$ = 0.03, so H2 was not supported. However, other not hypothesized relevant effects were found. The main effect for affect regulation strategy was significant, *F*(1,73) = 4.33, *p* = 0.04, $\eta_{p}^{2}$ = 0.06, with affect-improving participants, *M* = 2.54 (*SD* = 0.33), reporting significantly higher levels of anagram persistence than affect-worsening participants, *M* = 2.42 (*SD* = 0.24). The main effect of target’s feedback was not significant *F*(1,73) = 0.80, *p* = 0.37, $\eta_{p}^{2}$ = 0.01. Follow-up Fisher’s LSD *post hoc* comparisons showed that participants in the affect-improving condition had significantly higher anagram persistence, *F*(1,72) = 6.1, *p* = 0.02, $\eta_{p}^{2}$ = 0.08, Cohen’s *d* = 3.22, *M* = 2.62 (*SD* = 0.07), than affect-worsening, *M* = 2.41 (*SD* = 0.06), when receiving negative feedback, whereas for positive feedback the difference between affect improving, *M* = 2.45 (*SD* = 0.08) and affect worsening, *M* = 2.42 (*SD* = 0.05) was not significant, *F*(1,72) = 0.01, *p* = 0.63, $\eta_{p}^{2}$ = 0.003, Cohen’s *d* = 0.45.

### Self-rated Performance

**FIGURE 2:**
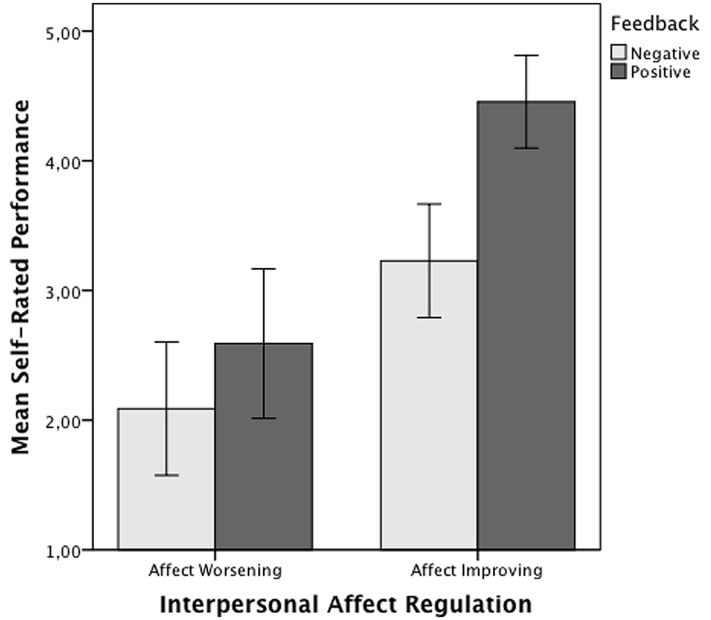
**Mean ratings of self-rated performance for interpersonal affect regulation strategy and feedback conditions**. Error bars represent 95% confidence interval.

For self-rated performance, the interaction between agent’s interpersonal affect regulation strategy and target’s feedback effect was not significant, *F*(1,73) = 2.64, *p* = 0.11, $\eta_{p}^{2}$ = 0.03, so H3 was not supported. The main effect for affect regulation strategy was significant, *F*(1,73) = 41.99, *p* = 0.001, $\eta_{p}^{2}$ = 0.36, with affect-improving participants, *M* = 3.7 (*SD* = 1.05) reporting significantly higher levels of performance than affect-worsening participants, *M* = 2.36 (*SD* = 1.17) (see **Figure 2**). The main effect of target’s feedback was also significant, *F*(1,73) = 14.26, *p* = 0.001, $\eta_{p}^{2}$ = 0.16, with participants receiving positive feedback, *M* = 3.45 (*SD* = 1.40), reporting higher levels of performance when compared with participants receiving negative feedback, *M* = 2.70 (*SD* = 1.14).

A follow-up analysis was conducted to test if the ego-depletion effect was responsible for the effects of interpersonal affect regulation and feedback on performance. The 2-way ancova for self-rated performance was repeated but with the behavioral and self-reported measures of ego-depletion included as covariates. The main effects of interpersonal affect regulation, *F*(1,73) = 26.75, *p* = 0.001, $\eta_{p}^{2}$ = 0.28, and feedback, *F*(1,73) = 9.23, *p* = 0.004, $\eta_{p}^{2}$ = 0.11, remained significant which indicated that ego depletion did not account for these effects.

## Discussion

Overall, our findings converge with a small number of relevant studies that have shown that the use of interpersonal affect-worsening regulation strategies contributes to agent’s ego-depletion, and have suggested that target’s feedback may act as a source of recovery from resources depleted by self-regulation effort during interpersonal affect regulation. Previous research has been based on either a survey methodology or has focused exclusively on affect-improving strategies. Unlike previous studies, our research experimentally analyzed the independent and combined effects of agents’ interpersonal affect regulation strategy use and targets’ feedback on agents’ resource depletion and self-rated performance. This occurred in the context of a realistic healthcare simulation in which doctors (medical students) provided healthcare consultations to patients (trained actors). Although hypothesized interaction effects between strategy use and feedback were confirmed only for emotional exhaustion, our results supply original evidence concerning the impact of target’s positive feedback on the experienced and expected emotional exhaustion of agents using affect-worsening strategies and on the consequence of target’s negative feedback for agent’s persistence on a self-regulation task, as well as consequences for performance.

For emotional exhaustion, our results supported the proposed interaction effect between the type of strategy used (i.e., whether it was aimed at improving or worsening affect) and the valence of the feedback. As expected, participants who tried to improve the affect of the patient and received positive feedback in return were less emotionally exhausted than participants who received negative feedback or who were required to worsen the patient’s affect. This result partially supports the idea that target’s feedback acts as a buffering factor against the self-regulation demands of interpersonal affect regulation. However, target’s feedback did not show a similar effect when participants tried to worsen target’s affect. Drawing on the conservation of resource model, the positive (lessening) effect of feedback on the agent’s emotional exhaustion is explained by the recovery of self-regulation resource through positive social relationships when targets positively reciprocate the agent’s attempts to improve their affect (Hobfoll, 1989; Brotheridge and Lee, 2002). However, when targets respond positively to the agent’s attempts to make them feel worse, the agents may perceive the feedback as undeserved or as inauthentic (e.g., motivated by the target’s fear of negative consequences). The absence of reciprocity and the perception of target’s feedback as unfair or insincere may impair the agent’s assessment of the interaction’s quality and reduce the potential for positive feedback to assist recovery. An analogous effect has been found in employee-client relationships wherein a client’s perception of inauthenticity in the employee’s emotional expression provokes a negative reaction in the client (Grandey et al., 2005). Under this condition, the target’s feedback may not be sufficient to restore the agent’s depleted self-regulation resource and other sources of recovery are needed (Schaufeli et al., 1996). Other sources of recovery (e.g., a colleague legitimating the use of affect-worsening), were not available in our study so affect-worsening was associated with resource depletion.

Our findings on expected emotional exhaustion (i.e., how exhausted participants thought they would be if they had to engage in similar patient interactions for a whole working day) did not show an interaction effect between the agent’s interpersonal affect regulation strategy and target’s feedback but did show main effects. Participants using affect-worsening reported higher expectation of being emotionally exhausted by future interactions than participants trying to improve target’s affect. Considering affect-worsening as a more depleting strategy, it is likely that participants estimation of the resource available after the actual interaction in the affect-worsening conditions was lower than for participants in the affect-improving conditions, and thus their expectation that future interactions would be exhausting was higher. In a similar vein, participants in the negative feedback conditions probably estimated that recovery of self-regulation resource in future interactions would be unlikely, increasing their estimation of emotional exhaustion following those interactions.

When agent’s ego-depletion was behaviorally measured by persistence on a subsequent self-regulation task, contrary to expectation the interaction between interpersonal affect regulation strategy and target feedback was not significant. However, participants using affect-improving strategies persisted significantly longer than participant trying to worsen the target’s affect. This suggests that affect-worsening requires more effort as a self-regulation process than trying to improve other’s affect. Research on individuals’ regulation of their own emotion has shown that some emotion regulation strategies dealing with negative emotion, such as emotional suppression (Richards and Gross, 1999), consume more self-regulation resource than other strategies trying to change the inner feelings and experience positive emotions. In a similar vein, it is possible that intentionally trying to induce negative feelings in other people requires agents to regulate their own negative emotions (e.g., guilt) or makes social coordination more psychologically taxing (Finkel et al., 2006). In the case of our study, it is also possible that participants in the affect-worsening condition had to make an effort to suppress their true feelings toward the confederate. Considering that participants had volunteered to take part in a study that simulated provision of medical care, these feelings are more likely to have been positive than negative. Suppression of positive feelings in order to deliver an affect-worsening strategy may therefore have consumed self-regulation resource, which would help explain why affect-worsening led to greater impairment of performance on a subsequent self-regulation compared with use of an affect-improving strategy.

Contrary to the expectation from a conservation of resource perspective, the effect of target’s feedback on subsequent task persistence was not significant. It is worth noting that negative feedback was associated with greater persistence when it followed affect-improving rather than affect-worsening, even though the valence of the feedback was incongruent with the valence of the affect regulation, which suggests a possible motivational effect. Recently, theorists advocating the strength model of self-regulation have posited that resource depletion after self-regulation is only partial and temporary, with a reservoir of resource remaining for future demands of self-regulation (Baumeister, 2014). Because the modification of the model assumes a certain amount of self-regulation resource is available for future acts of self-regulation, it has been proposed that changes in self-regulation performance may have to do with motivational factors (Job et al., 2010; Inzlicht and Berkman, 2015). The unexpected result in our study may indicate that participants using affect-improving interpreted the target’s negative feedback as a sign of poor performance during the interaction and thus tried to compensate for it by over-performing in the subsequent task. This motivational effect may have overcome the partial depleting effect arising from the interpersonal regulation of affect.

Our findings for the different measures of ego-depletion show that trying to worsen others affect is a more depleting self-regulated behavior than trying to improve others affect. The results also suggest that the recovery effect of targets’ feedback on resource depletion are not straightforward because: for affect-worsening, positive feedback had no effects for experienced emotional exhaustion or anagram persistence but a positive effect for expected emotional exhaustion; while for affect-improving, negative feedback had a negative effect for experienced and expected emotional exhaustion, but a positive effect for anagram persistence. These differences may be explained in terms of the feedback’s impact on social coordination and on motivation. Positive feedback in response to affect-worsening may increase agents’ perception of the effort that will be required for social coordination if agents decode the response as a sign either that targets’ affect has not changed (i.e., the regulation goal has not been achieved) or that targets have not recognized they are being alerted to a problem that requires attention. In this circumstance, positive feedback could be ineffective in mitigating depletion of self-regulation resource. Negative feedback in response to affect-improving may have an immediate depleting effect but it may also motivate agents to improve their performance on a subsequent task by increasing their persistence in order to compensate the perception that they have performed poorly during the interaction (as indicated by the self-reported performance data).

The study also shows that interpersonal affect regulation strategies are relevant for organizational outcomes that arise from service encounters to the extent that individual’s ratings of their performance were explained by the type of strategy they used to regulate the target’s affect. To the author’s knowledge previous research has not investigated the relationship between interpersonal affect regulation and perceived performance. Unexpectedly, the interaction effect between regulation strategy and target feedback was not significant, but their main effects were significant. Use of affect-improving strategies and positive feedback from targets were independently related to higher self-rated performance for the interactions when compared with affect-worsening and negative feedback, respectively. Follow-up analysis showed that these differences could not be explained by the effects of interpersonal affect regulation on ego-depletion as expected from a social coordination perspective (Finkel et al., 2006). A plausible alternative explanation is that agents use the valence of the target’s feedback as an indicator of personal performance, which they compare against organizational and occupational norms for emotion regulation. This explanation is described in more detail below.

Previous research has shown that regulation of affect in work settings is not left solely to the employee’s personal criteria (Denison and Sutton, 1990); it is also bound to emotional norms defined in large part by the specific occupational groups or teams in which the role occurs (Smith and Kleinman, 1989; Martínez-Iñigo et al., 2009; Diefendorff et al., 2011). Regulation of emotions to comply with organizational or occupational norms is one of the elements that defines effective performance in different job roles (Yanay and Shahar, 1998). These norms shapes employee’s regulation of their own and client’s affect in a way that facilitates the satisfaction of clients’ demands in a professional fashion (e.g., high quality health care delivery). Insufficient or inappropriate emotion regulation can be considered as a sign of low professionalism (Smith and Kleinman, 1989; Denison and Sutton, 1990; Yanay and Shahar, 1998; Harris, 2002; Diefendorff et al., 2011).

Emotion norms constitute a standard against which employees compare their performance, so the use of an interpersonal affect regulation strategy to meet those norms can be related to their perceived performance. Although exceptions can be found for specific occupations (e.g., debt collectors) or specific situations within a job (e.g., interaction with abusive clients), emotion regulation at work it is generally governed by a “service with a smile” norm that prescribes the expression of positive emotions and the suppression of negative emotions. In the case of interpersonal affect regulation this will involve a preference for strategies that try to improve client’s affect rather than worsening their affect. Thus, it is plausible that participants acting as doctors will normatively rate their performance higher if they try to improve targets’ affect compared to those who try to worsen targets’ affect.

Feedback from clients is key information that employees can use to assess their performance (Brotheridge and Grandey, 2002; Brotheridge and Lee, 2002). Previous research on employee’s regulation of their own emotions has shown that the negative impact of inefficient emotion regulation on self-efficacy increases when it is met with annoyance by clients (Brotheridge, 1999). In line with this idea that feedback provides information about performance, it is plausible that participants acting as doctors will rate their performance lower when receiving negative feedback from “patients” compared with those who receive a positive response.

Overall the study contributes to an understanding of the consequences of emotion regulation by providing evidence on the depleting effects of interpersonal emotion regulation during service encounters. The number of studies of interpersonal emotion regulation is tiny in comparison with research on the intrapersonal dimension. The study suggests that interpersonal affect regulation is relevant not only for employee’s well-being but also for organizational outcomes that are reliant on job performance. Our results are especially relevant for interactions with clients that occur in a healthcare setting. The use of an experimental design based on healthcare interactions with simulated patients supplies robust evidence concerning relationships between variables of interest in this setting.

Besides the theoretical relevance of the study, the findings have some practical implications for healthcare organizations. Our results showed that interpersonal recovery processes are sometimes unable to compensate for the depleting effect of interaction with patients. Moreover the necessity to interact with people who are dealing with discomfort or suffering makes it inevitable that healthcare professionals will face some amount of negative feedback even when they try to improve how patients feel (Demerouti et al., 2001). Under such circumstances individuals may have to turn to the organization in search of compensation (Schaufeli et al., 1996). Previous research has shown that organizational failure to reward employee’s self-regulation effort is positively related to employee’s use of counterproductive behaviors (Bechtoldt et al., 2007). On the contrary, explicit acknowledgment of the employee’s effort to self-regulate their emotions has been shown to reduce its negative impact on employee’s satisfaction (Grandey et al., 2013). Healthcare organizations need to be aware that the requirement to regulate patient’s affect adds to health professional’s work demands, and should address this impact in their human resource policies and practices. Promoting resource recovery from other sources such as colleagues, teams or supervisors could prevent states of depletion that are recognized as a component of burnout in health professionals (Maslach, 1993). Grandey et al. (2012) study of health professionals regulation of their own emotions found that emotional demands from the interaction with patients were not related to the health professional’s burnout when they were integrated in work units with a climate of authenticity that encouraged and supported authentic emotional expression among team members. The development of a protective group climate could be beneficial to prevent deterioration in the well-being of health professionals and its negative impact on organizational outcomes through lower job performance, absenteeism, or turnover (Grandey et al., 2012).

Health professional’s response to patients’ negative feedback can also contribute to reducing its negative impact on well-being. Health professionals can perceive patient’s negative feedback as an ego threat (Baumeister et al., 1993) and respond defensively through self-enhancement behaviors (e.g., self-serving attributions). Despite being a common response to sustain individuals’ positive view of themselves, self-enhancement responses can have negative consequences for individual’s self-control and well-being (Baumeister et al., 1993). Schmeichel and Vohs (2009) found that developing self-affirmation responses to ego threats prevents self-protective responses and counteracts ego-depletion by promoting a more global and abstract definition of a particular event (high-level construal). High-level construal of an event (e.g., patient’s negative feedback) focuses on the abstract meaning of the event, and weighs its relative relevance for the achievement of long-term goals, instead of paying attention to the immediate feelings (Fujita et al., 2006). Developing the health professional’s skills to frame patient’s negative feedback as useful informative events in the overall process of improving the patient’s health could contribute to reducing defensive responses and their impact on ego-depletion.

Previous research has also shown that training employees in cognitive change and attentional deployment techniques can improve their effectiveness in regulating their own emotions and reduce the use of strategies related with poorer well-being (Hülsheger et al., 2015). Use of other cognitive techniques, such as mindfulness, have shown similar effects (Hülsheger et al., 2013). Training health professionals to reappraise personally distressing aspects of patient’s behaviors or to turn their attention toward more pleasant thoughts may increase their motivation to enact more positive interpersonal affect regulation strategies (affect-improving strategies) and reduce some of the negative consequences of patient’s negative feedback (Crego et al., 2013). Healthcare organizations and educational institutions should consider integrating these elements in their training plans and curricula to help equip health professionals with the emotion regulation competencies required to maintain their own mental health and deliver best quality care to their patients at the same time.

### Limitations and Future Directions

Although the present findings provide valid evidence on the combined effects of agent’s use of interpersonal affect regulation and target’s feedback on agent’s ego-depletion and self-rated performance, a number of limitations can be identified. Firstly, the use of a laboratory study can threaten the generalizability of results. Although the experiment used a highly realistic setting and scenario and professional actors were trained to perform as patients, differences between the experimental simulation and real healthcare consultations could have affected the results. The small number of simulated patients and the brief amount of time that participants spent interacting with each confederate needs to be considered. The duration of the two simulated consultations was slightly under the average consultation time in primary care settings (Martín and Jodar, 2008). General practitioners also have greater variability in the amount of time they typically spend with patients and they attend to a greater number of patients than in our study. Future studies based on quasi-experimental or experience-sampling designs should assess how the number and duration of interactions with patients affects healthcare professional’s ego-depletion.

Secondly, the use of an experimental design eliminated other potential sources of recovery that would usually be available in real healthcare settings. For instance, the presence of other professionals (e.g., nurses) in the surgery or the opportunity for a doctor to debrief with a colleague after the patient consultation might have consequences for a doctor’s level of ego-depletion.

Thirdly, the study had to make conjectures about variables that could have accounted for unexpected results. Future research should empirically test the role of these variables. In particular, studies should measure the effects of positive feedback on social coordination when agents try to worsen targets’ affect and empirically test its consequences for emotional exhaustion. The proposed motivational effect of negative feedback in response to affect-improving and its consequence for subsequent performance also needs empirical confirmation. Inclusion of objective measures would also supply additional evidence concerning the relevance of interpersonal affect regulation strategies for performance-related organizational outcomes.

## Conclusion

The findings from this study provide evidence that using interpersonal affect regulation strategies has consequences for the regulator’s resource depletion and performance. They also indicate that positive responses from the person that the affect regulation is aimed at can help ameliorate depleting effects. More importantly, our results suggest that the combination of the interpersonal affect regulation strategy that people use and the response they receive from their interaction partner can reliably predict their emotional exhaustion. The results are relevant for healthcare organizations and suggest that medical professionals need to be accomplished in delivering “good” and “bad” affect, as well as the more widely recognized good and bad news, to patients. Future research should step further in examining the processes that link different sequences of interpersonal affect regulation and feedback to employee’s well-being and performance to deepen our understanding of the interpersonal dimension of emotion regulation and its practical relevance.

## Conflict of Interest Statement

The authors declare that the research was conducted in the absence of any commercial or financial relationships that could be construed as a potential conflict of interest.
