# Corrigendum: Using interpersonal affect regulation in simulated healthcare consultations: an experimental investigation of self-control resource depletion

**DOI:** 10.3389/fpsyg.2018.01253

**Published:** 2018-07-10

**Authors:** David Martínez-Íñigo, Francisco Mercado, Peter Totterdell

**Affiliations:** ^1^Department of Psychology, Universidad Rey Juan Carlos, Madrid, Spain; ^2^Department of Psychology, Sheffield University, Sheffield, United Kingdom

**Keywords:** affect regulation, self-regulation, conservation of resource, ego depletion, performance, emotion, healthcare

In the original article, we neglected to include the co-funder **European Regional Development Fund: ERDF**. The authors apologize for this error and state that this does not change the scientific conclusions of the article in any way.

The original article has been updated.

## Conflict of interest statement

The authors declare that the research was conducted in the absence of any commercial or financial relationships that could be construed as a potential conflict of interest.

